# Kinesiology Taping Does Not Affect Tarsal Joint Motion During Selected Exercises in Dogs †

**DOI:** 10.3390/vetsci12050439

**Published:** 2025-05-03

**Authors:** Rebecca C. Noel, Leann M. Shaw, Nicholas H. Millis, Krysta Janas, Darryl L. Millis

**Affiliations:** 1 Metairie Small Animal Hospital, New Orleans, LA 70005, USA; bnoeldvm@gmail.com; 2 VCA California Veterinary Specialists, Murrieta, CA 92563, USA; leannshawdvm@gmail.com; 3 Veterinary Care and Specialty Group, Chattanooga, TN 37408, USA; nmillisdvm@gmail.com; 4 BluePearl Pet Hospital Gwinnett, Lawrenceville, GA 30043, USA; krysta.janas@gmail.com; 5 Department of Small Animal Clinical Sciences, College of Veterinary Medicine, University of Tennessee, Knoxville, TN 37919, USA

**Keywords:** gait analysis, kinematic gait evaluation, kinesiology tape, kinetic gait evaluation

## Abstract

Background: Use of kinesiology taping has increased in veterinary medicine, yet there are few studies of its effects. The purpose of this study was to evaluate the effect of kinesiology tape placed on the dorsal surface of the tarsus on kinetic and kinematic gait characteristics while performing selected exercises in dogs using 3D motion capture and force platform analysis of gait. Methods: Ten clinically normal, healthy, adult mixed-breed dogs were recruited for this study. Reflective markers were applied to the skin of the left and right pelvic limbs of each dog. Eight infrared cameras were positioned around a 13 m platform containing a force platform. Dogs were walked, trotted, and led over cavaletti rails with and without kinesiology tape extending from the distal tibia, crossing the cranial surface of the tarsus, and extending to the dorsal surface of the metatarsals. The evaluations were repeated 2 h later. Maximum flexion and extension in the sagittal plane and maximum angular acceleration and velocity were measured at the tarsus and stifle for each dog. Peak vertical (Z_Peak_), braking (Y_A_), and propulsion (Y_B_) forces were determined as a percentage of body weight while walking and trotting. Results: Ground-reaction forces were greater at the trot compared to the walk, and stifle and tarsal flexion were greater with stepping over cavaletti rails as compared to walking and trotting. There were no differences in kinematic or kinetic measurements related to the kinesiology tape application, time, or exercise being performed. Conclusions: Kinesiology tape had no effect on tarsal joint gait characteristics while walking, trotting, or stepping over cavaletti rails when applied to the dorsal tarsus of normal dogs.

## Simple Summary

Kinesiology taping has increased in use in human and veterinary medicine, both for athletic performance and treatment of various musculoskeletal and neurologic conditions. Studies in people suggest a mild effect for many uses. There are very few studies regarding kinesiology taping in veterinary medicine. Prospective evaluation of its effect on gait and mobility is essential to evaluate the efficacy of kinesiology taping. The objective of this study was to evaluate the effect of kinesiology taping applied to the tarsal joint and its effect on selected exercises in dogs using kinetic and kinematic gait analysis. In normal dogs, kinesiology taping had no effect on weightbearing forces at a walk and trot, and motion of the stifle and tarsal joints while walking, trotting, or stepping over cavaletti rails. Kinesiology taping should be assessed in other joints and in dogs with neurologic or orthopedic conditions.

## 1. Introduction

Kinesiology taping has increased in popularity both in human and veterinary applications since its inception in the 1970s. Today, it is commonly seen in athletes of numerous species in various activities. Application of kinesiology tape to the skin reportedly targets sensory receptors of the cutaneous skin, lifts tissues to provide lymphatic drainage, and improves circulation with simultaneous analgesia via nociceptive transmission inhibition [1]. There may be a benefit in using kinesiology tape for pain management [2,3,4,5], although the effects seem to be mild in musculoskeletal conditions, including osteoarthritis pain [6,7,8]. Additionally, kinesiology tape is postulated to stimulate pathways related to the mechanoreceptive and proprioceptive pathways, thereby modifying joint kinematics [9]. In humans, it is also used to improve athletic performance despite a lack of literary support [10]. However, most studies indicate negligible benefit for muscle strengthening in healthy adult humans [11,12], and there may be few benefits regarding athletic performance in healthy individuals [10]. Some studies have suggested an improvement in muscle fatigue [13,14,15], while others have shown no improvement in muscle strength or athletic performance [3,10,16,17]. Further, kinesiology taping may have limited effects to reduce swelling after an acute ankle sprain [18,19].

In veterinary medicine, kinesiology tape is used in horses to treat muscular conditions, fascial restrictions, and postural imbalance, although there is little evidence of beneficial effects [9,20,21,22]. Ramon et al. demonstrated decreased peak vertical force and limited flexion of the fetlock in horses during the swing phase of gait with kinesiology tape applied [22]. A study of the effects of kinesiology tape applied to the forelimbs of horses showed no effect on forelimb kinematics or muscle EMG activity [23]. However, application to the abdominal muscles increased craniocaudal activity of the body [24].

There is very little evidence regarding kinesiology tape application techniques or how it affects joint motion and weight bearing during simple exercises in normal dogs. The authors have used kinesiology tape on several dogs with various pathologies and perceived efficacy in terms of increased joint motion. However, the magnitude of effect and the duration of action have not been evaluated under controlled conditions with objective outcome measures in normal dogs. With the advent of veterinary-specific kinesiology tape, manufacturers claim the tape can last for 5 days and can be used to treat conditions ranging from muscle injury, inflammation, tendonitis, desmitis, and increased joint range of motion (RockTape, https://rocktape.com.au/canine/ (accessed on 3 March 2025)).

The purpose of this study was to investigate gait changes following the application of kinesiology tape on the dorsal canine tarsus. Additionally, tape longevity and application protocol were evaluated. We hypothesized that there would be increased range of motion and decreased peak vertical force between the taped limbs of each dog at the walk, trot, and while walking over cavaletti rails.

## 2. Materials and Methods

**Participants:** The study protocol was approved by the Institutional Animal Care and Use Committee at the University of Tennessee, and written informed consent was obtained from owners. Ten client-owned dogs were included in the study reported here. Weight, age, and breed were recorded. An initial baseline evaluation was performed, including orthopedic, neurologic, and general physical examinations. Individuals were excluded for any history of significant mobility disorder, obvious visual lameness, greater than 10% difference in peak vertical force between forelimbs or between hind limbs at a walk and a trot, if gross joint instability was present, or if there were any other clinically abnormal findings on physical examination. Dogs were between 2 and 10 years of age and weighed between 15 and 50 kg.

**Kinematic Data Collection**: A 3-dimensional testing space measuring 3 × 1 m was established on a 13 m walkway centered over the force platform. A right-handed orthogonal global coordinate system was established within the 3D testing space with 0,0,0 (X, Y, Z) located on the force platform. On each data-collection day, 8 high-speed data-capture cameras (250 Hz Vero cameras, Vicon Motion Systems Inc., Centennila, CO, USA) were calibrated to the testing space using an active calibration wand (Vicon Motion Systems Inc., Centennila, CO, USA). Markers were tracked, and motion capture data were recorded using commercial software (Vicon Nexus version 2.11, Vicon Motion Systems, Inc.).

**Figure 1:**
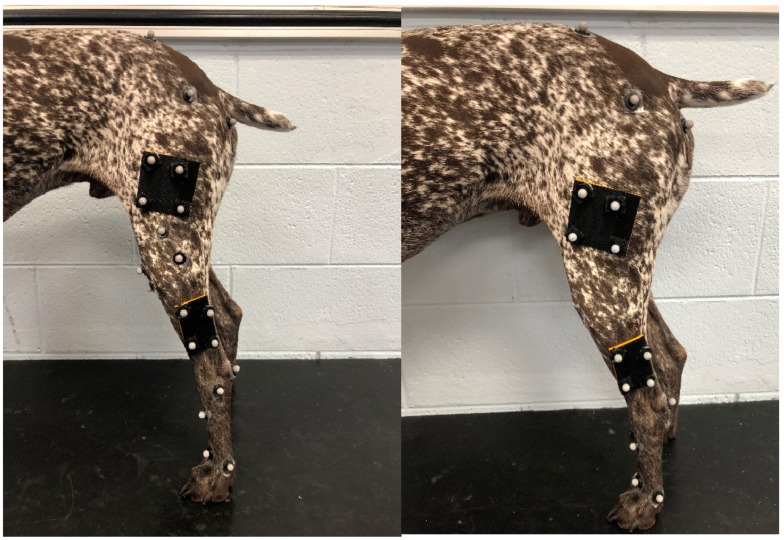
Anatomic (calibration) marker set (**left**) and tracking marker set (**right**).

**Figure 2:**
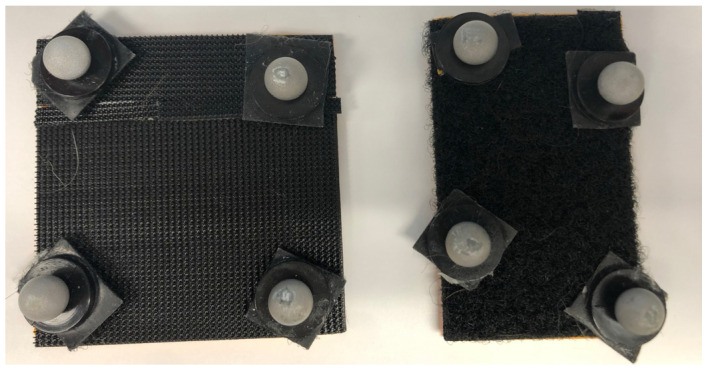
Rigid cluster design using a 3D-printed curved board. Each cluster was arranged in a rhomboid shape with an approximate distance of 2.5 cm separating each marker from the center of the sphere. Markers were arranged in a non-colinear fashion.

Motion capture began with a static calibration with 10–15 frames of data captured, with 23 reflective, spherical markers on each pelvic limb (46 total markers) placed on anatomic landmarks or as rigid femur cluster markers on femurs and tibias as described by Fu et al. (Figure 1) [25]. We used multiple rigid cluster design methods based on experience from animal and human biomechanic studies. A 2 × 2 cm and a 3 × 3 cm 3D-printed arched boards using a 20% infill were constructed for application of cluster markers (Figure 2). The boards were covered with Velcro, and the markers were affixed. The arched boards and reflective markers were then affixed to animals using double-sided medical tape and GLUture (Zoetis Inc., Kalamazoo, MI 49007, USA), if necessary. Hair in the area was clipped if more than 0.5 cm in length.

Following the calibration trial, seven markers over anatomic landmarks were removed for the remaining motion trials (Table 1). These markers were virtually reconstructed from the initial static trial using Vicon Procalc software, version 1.5 (Vicon, Colorado) [25,26]. Each dog was walked, trotted, and walked over cavaletti rails four times with the cluster markers in place. The order of data collection was randomized among the three exercises. Data were acquired from the right and left sides separately.

**Figure 3:**
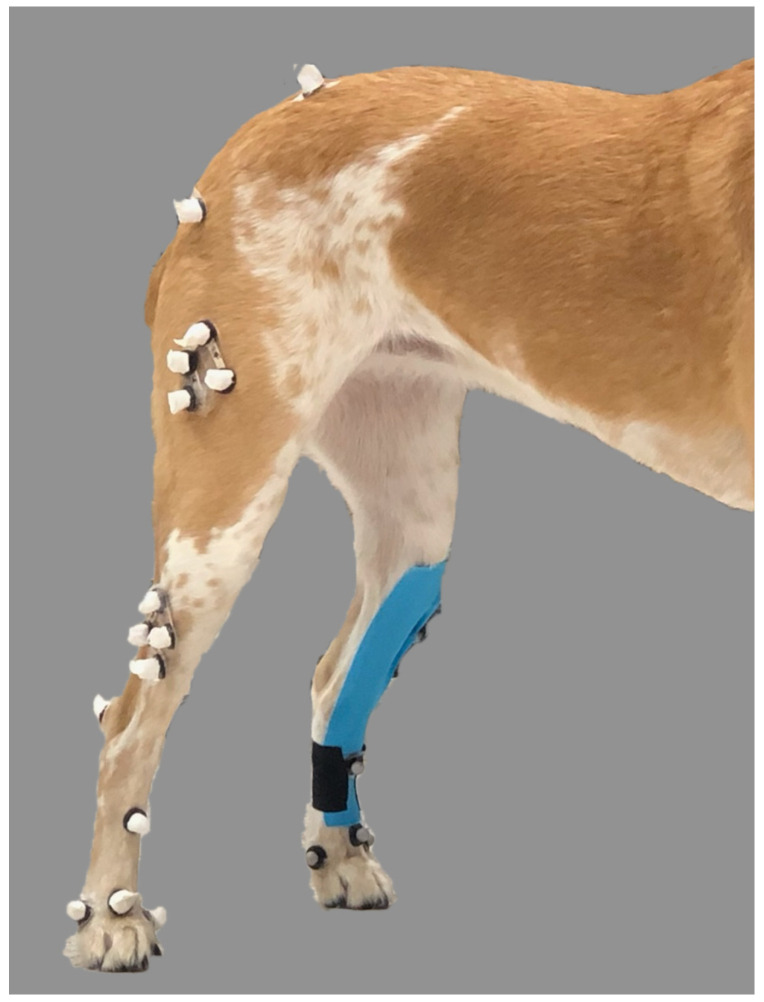
Instrumented dog with kinesiology tape applied.

After these trials were captured, kinesiology tape was applied to the dorsal surface of a randomly selected tarsus from the distal tibial tuberosity to the distal metatarsus. The tape was applied by stretching the tape to 25% of the unstretched tape length (to apply under tension) and placed from proximal to distal (Figure 3). We found it necessary in initial attempts to apply a 3 cm-wide anchoring piece (not stretched) at the proximal and distal ends to help hold the tape in place, according to the manufacturer’s instructions (RockTape, Morley, WA, USA). After application, the tape was rubbed for 3 min to activate the adhesive prior to the acclimation period. Dogs were allowed to acclimate to the tape during 5 min of free exercise. Dogs were then walked, trotted, and walked over cavaletti rails to collect kinetic and kinematic data as described, and data were collected 5 min and 2 h after tape application. Cavaletti height was based on the size of the dog, with the cavaletti rail height placed at the junction of the lower and middle third of the antebrachium.

Joint coordinate system: The local coordinate systems (LCS) specific to each segment were designed similarly to Fu et al. [25], with the exception of the foot. The LCS of our foot originated at the caudal aspect of the calcaneus (CALC), with the unit vector of the z-axis defined by the vector between the second and fifth distal metatarsals, the x-axis unit vector was defined by the vector from the caudal calcaneus travelling distally to bisect the z-axis vector. The y-axis unit vector was defined as a cross product of the X and Z vectors. All joint angles were converted to complementary angles, as in the modeled papers [26,27,28,29]. Kinematic data were gap-filled, filtered, and smoothed using Butterworth filtering. Data collected included tarsus joint angles in the X, Y, and Z planes, including peak tarsus and stifle joint flexion and extension, angular velocity, and angular acceleration.

Kinetic Data Collection:

Ground-reaction forces were determined using a force platform (AMTI OR6-6, Watertown, MA, USA) and quadruped software (Acquire version 7.33, Vicon, Centennial, CO, USA). Dogs were walked and trotted over the platform between 0.7 and 1.2 m/s and 1.7 and 2.1 m/s, respectively, with an acceleration of ±0.5 m/s^2^. Speed and acceleration were measured using five photoelectric cells mounted 50 cm apart at a height of 58 cm within the testing space. Trials were included for analysis if ipsilateral forelimb and hindlimb strikes occurred, velocity and acceleration were within the described parameters, and there were no sudden changes in limb, body, or head motion. Four valid right and left ipsilateral limb strikes were obtained, and the means of the four trials were calculated for each parameter for each side. Peak vertical (Z_Peak_), braking (Y_A_), and propulsion (Y_B_) forces were determined as a percentage of body weight. Visual examination of the graphical representation confirmed the validity of trials. Kinetic data were collected before tape application and 5 min and 2 h after application.

Kinetic and Kinematic Data Analysis:

A three-way repeated measures ANOVA (SAS, Cary, NC, USA) was performed on ground reaction forces, angular acceleration, angular velocity, and maximum and minimum joint angles of the tarsus and stifle. Comparisons between the taped and untaped limbs, as well as comparisons between exercises (walking, trotting, and cavaletti poles), and exercise × time interaction were performed.

## 3. Results

### 3.1. Participants

A total of 10 dogs participated in the study. Breeds included German Shepherd, Standard Poodle, Golden Retriever Mix, Boxer mix, Great Dane, Weimaraner mix, English Retriever Spaniel, and mixed breed dogs. The average age of participants was 6.2 years (range 2 to 10), and the average weight was 25.8 kg (range 17.4–40.4). The body condition score of dogs was 4–6 out of 10. Four dogs had the tape applied to the left tarsus, and six dogs had the tape applied to the right tarsus.

All dogs had short-to-medium length hair; dogs with medium-length hair were clipped for marker application. One dog (German Shepherd) experienced minor dermatitis from clipping. It was treated with topical dilute chlorohexidine and resolved in 5 days without complication. No other complications occurred.

### 3.2. Kinesiology Tape Longevity

**Figure 4:**
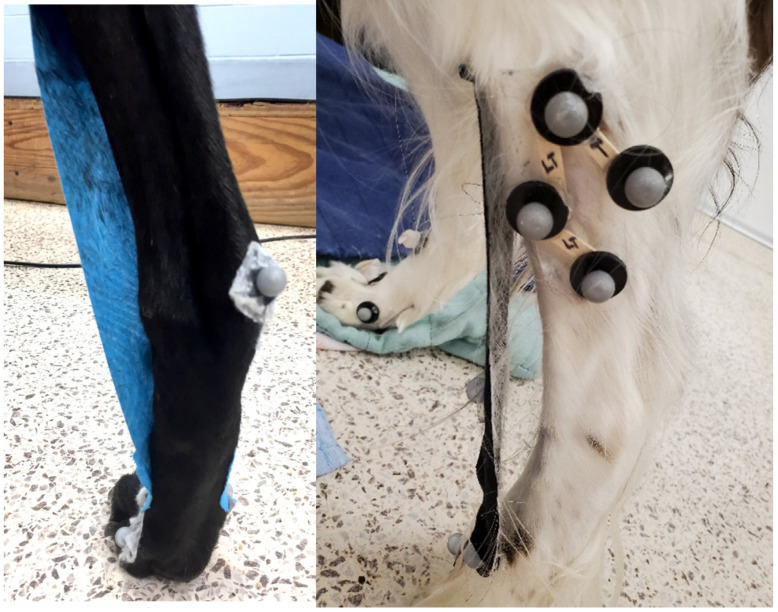
Kinesiology tape failure over the tarsocrural joints.

**Figure 5:**
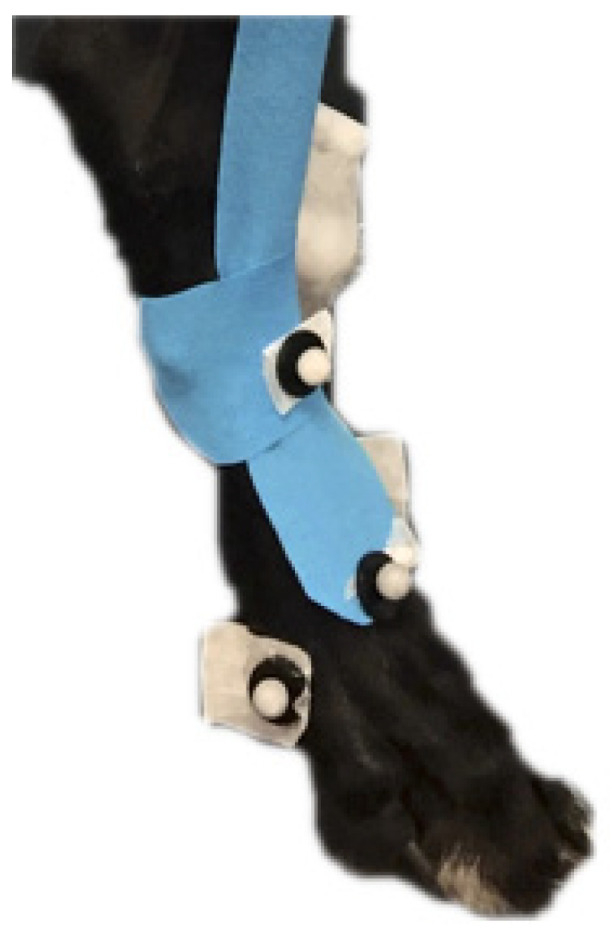
Additional tape added perpendicular to the original tape to hold it in place. Each subject required this modification by the 2-h test mark or earlier.

The kinesiology tape either fell off or required additional support pieces applied within 2 h (Figure 4). In most dogs, the tape loosened within 15 min. Additional support pieces were placed perpendicularly on the limb depending on the site of tape failure (Figure 5). Failure occurred most commonly at the tarsocrural joint, likely due to the high motion and angularity in this area. Other sites of failure included the proximal and distal ends of the tape.

### 3.3. Kinematic Gait Evaluation

**Figure 6:**
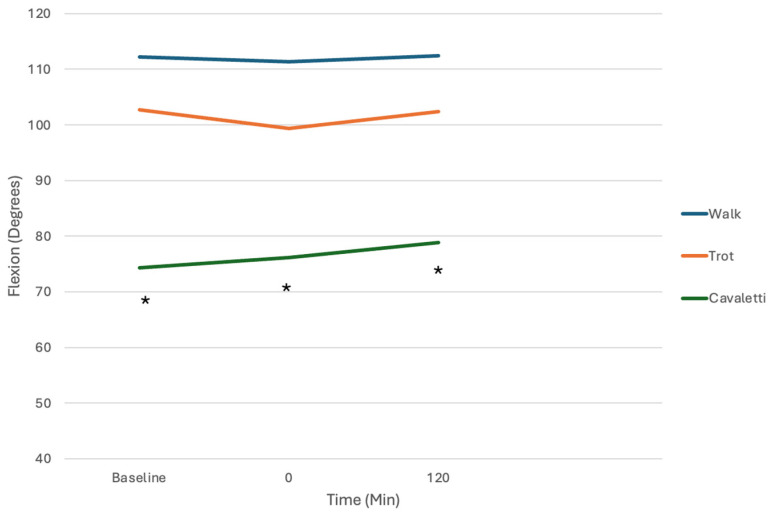
Stifle flexion at a walk, trot, and stepping over cavaletti rails before and after kinesiology tape application. Flexion was significantly greater with stepping over cavaletti compared with walking and trotting, but there were no differences with kinesiology tape application (* indicates significance between walking vs. cavaletti and trotting vs. cavaletti).

**Figure 7:**
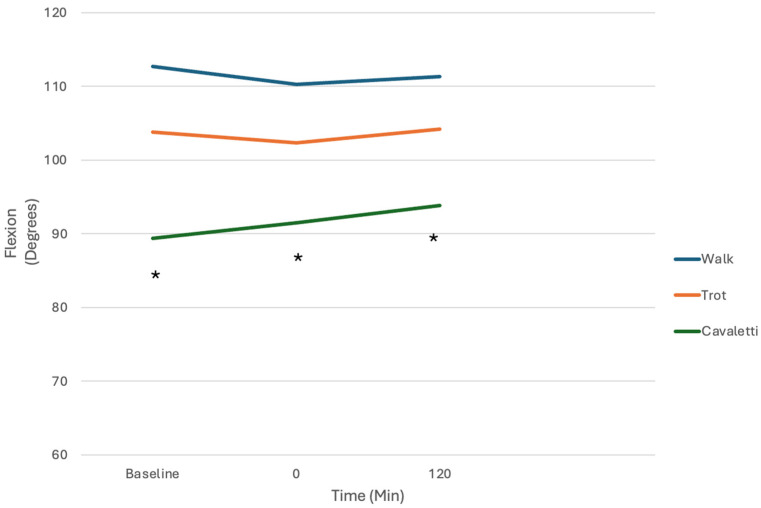
Tarsal flexion at a walk, trot, and stepping over cavaletti rails before and after kinesiology tape application. Flexion was significantly greater for stepping over cavaletti rails compared with walking and trotting, but there were no differences with kinesiology tape application (* indicates significance between walking vs. cavaletti and trotting vs. cavaletti).

There were no significant differences among any of the comparisons, except for significant changes in angular acceleration and angular velocity of joint motion and tarsal and stifle flexion among exercises (Table 2 and Table 3, Figure 6 and Figure 7). Flexion was greatest with cavaletti rail walking, followed by trotting and walking. The presence of kinesiology tape application had no effect on altering the measured variables during a particular exercise, however.

### 3.4. Kinetic Gait Evaluation

**Figure 8:**
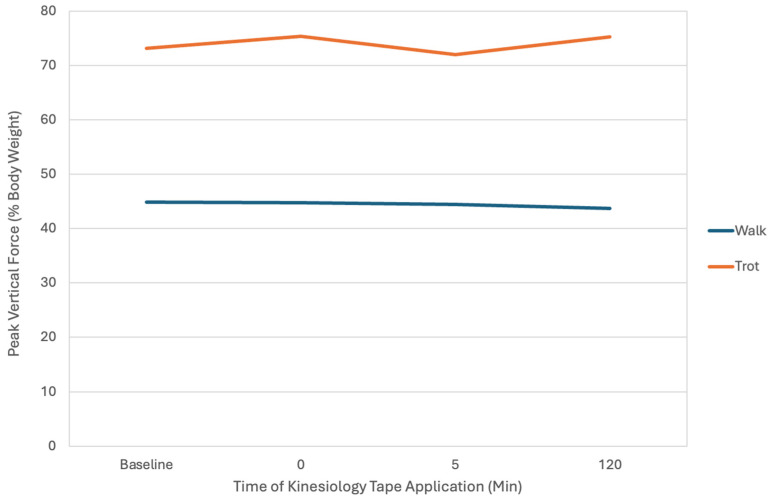
Peak vertical force of dogs walking and trotting before and after kinesiology tape application. Peak vertical force was significantly greater with trotting vs walking, but there were no differences with kinesiology tape application.

Three-way repeated measures of ANOVA showed no statistical difference between the taped and untaped limbs between limbs, exercise × time, or other comparisons listed in Table 4. There were differences in mean ground reaction forces regarding exercises, with Z_Peak_ and Y_APeak_ being greater with trotting (Figure 8).

## 4. Discussion

Based on the results reported here, the application of kinesiology tape to the canine tarsus had no significant effect on tarsal or stifle joints regarding gait or performance of selected exercises. The tape had no effect on kinematic joint variables in the tarsus or stifle, similar to the effect of kinesiology taping of the forelimb in horses [23], nor any effect on measured ground reaction forces at the walk or trot, unlike a study of tape application to equine fetlocks [22]. Therefore, our original hypotheses postulating increased range of motion and decreased peak vertical force were rejected.

Studies in people have also found little effect of kinesiology tape on joint motion [30]. However, improvement in muscle strength has been reported in people undergoing kinesiology taping for knee osteoarthritis, muscle fatigue, and shoulder function during performance activities [14,31,32], while other studies have shown no effect [33,34,35]. Although improvement of joint proprioception has been a suggested benefit of kinesiology taping [36,37] and may be beneficial in those with poor proprioception [38], this may not occur in normal patients [39,40,41].

A secondary aim of this study was to evaluate kinesiology tape longevity based on the manufacturer’s claims that it can be worn for 5 days. The tape was applied per the manufacturer’s instructions. In this study, the kinesiology tape did not adhere to the limb as long as expected. All dogs required tape modification within 2 h of application. However, this may be due to the high mobility of the canine tarsus and may not reflect inherent properties of the tape itself or the tape application protocol. It is possible the tape may perform better on other joints or on dogs that are not performing exercises with great joint motion after tape application. It is also unknown if the additional pieces added to maintain tape position (added perpendicularly to the original piece) altered joint motion. Further studies using tape on other joints may produce different results and provide more longevity of the tape.

Based on the results of the study presented here, kinesiology tape does not alter gait or performance of the tested exercises in clinically normal dogs. The tape did not affect any of the measured parameters when compared to the untaped contralateral limb. This is similar to the effect of kinesiology taping of the forelimb in horses and in back flexion and extension, which found no effect on forelimb joint or back kinematics [9,23]. However, the lack of effect of kinesiology taping on ground-reaction forces in dogs differs from a study of tape application to equine fetlocks, which found decreased ground reaction forces [22]. Although we found no effects in our study of normal dogs, studies of kinesiology taping effects in dogs with pathology, such as osteoarthritis, should be considered [42].

Studies evaluating tape application methods may also be warranted. Although some studies have suggested that tape tension and the direction of tape application may make a difference [43,44,45], others have shown that the direction of the tape may not make a difference regarding athletic performance [46,47,48]. Changes in application, such as clipping the hair over the intended area of all study dogs, may help increase contact between the adhesive and skin. Also, tapes from different manufacturers exhibit different characteristics regarding adherence [49,50,51].

Limitations in this study include a small sample size and difficulties in the application of the kinesiology tape and tape longevity. Skin movement about markers is also a limitation of the kinematic evaluation of gait. However, the techniques used in this study allowed noninvasive collection of data and used no radiation equipment that has been used in other studies, such as fluoroscopy, to limit radiation exposure. In addition, the use of the virtual markers used in the study reported here has been shown to reduce the effects of marker motion artifact. Each dog served as its own untaped control, making any differences in joint motion a result of kinesiology tape application consistent.

In our original hypothesis, we believed that dogs would have altered tarsal joint movement, and we also believed that this effect would be less as the dogs became accustomed to wearing the tape. That was the logic behind repeated evaluation over 2 h or longer. However, because there were no immediate differences in joint motion right after tape application, along with difficulties in maintaining tape in position, we were unable to evaluate the dogs longer than 2 h after tape application.

To our knowledge, this is the first study to evaluate the effect of kinesiology tape on gait characteristics in normal dogs. Our study of the effects of kinesiology tape on dogs is important to further understand any impacts on clinical patients. Kinesiology tape is increasingly used in veterinary practice, likely because of its impact on human sports medicine. Systematic scientific studies are warranted to understand the true effectiveness of the tape. Future research to evaluate the use of kinesiology tape on other joints, such as the stifle, coxofemoral, and cubital joints, as well as the spinal column, is warranted in clinically normal dogs and dogs with various orthopedic or neurologic conditions.

## 5. Conclusions

In conclusion, kinesiology tape applied to the dorsal canine tarsus had no significant effect on gait or the completion of selected exercises. The tape had no effect on measured kinematic or kinetic variables. The tape, as applied according to the manufacturer’s instructions, did not last beyond 2 h. The tape application protocol should be revisited if subjects are asked to perform rehabilitation exercises over a period of time. Additional studies evaluating tape application, tape longevity, and tape effects on other joints during motion are also warranted.

## Figures and Tables

**Table 1:** List of anatomic markers, ** indicates markers that were removed after kinematic calibration. The greater trochanter was shared between the pelvis and femur segments for calibration but was only used for the pelvis during data acquisition (tracking).

Pelvic Limb Segments	Marker Location (Right and Left Limb)
Pelvis	Iliac wing
	Ischium
Femur	Greater trochanter
	Lateral epicondyle **
	Medial epicondyle **
Tibia	Fibular head **
	Proximal tibial crest **
	Distal tibial crest **
	Lateral malleolus **
	Medial malleolus **
Foot	Point of calcaneus
	Metatarsophalangeal joint 2
	Metatarsophalangeal joint 5
	Proximal tarsometatarsal joint
	Distal tarsometatarsal joint
Cluster Markers (4 each)	Femoral cluster
	Tibial cluster

**Table 2:** Summary of three-way repeated measures ANOVA of tarsal kinematic measurements.

Tarsus Kinematic Variables (*p* Values)				
Comparison	Angular Acceleration	Angular Velocity	Maximum Flexion	Minimum Flexion
Affected vs. Unaffected Leg	0.5042	0.5418	0.6946	0.7511
Exercises	0.0004	0.0001	0.1554	<0.0001
Leg vs. Exercises	0.7955	0.4757	0.3118	0.4251
Time	0.7509	0.9452	0.9960	0.3644
Leg vs. Time	0.9848	0.9041	0.5699	0.8166
Exercises vs. Time	0.0861	0.3684	0.3258	0.0596
Leg vs. Exercises vs. Time	0.2671	0.5127	0.8765	0.9506

**Table 3:** Summary of three-way repeated measures ANOVA of stifle kinematic measurements.

Stifle Kinematic Variables (*p* Values)				
Comparison	Angular Acceleration	Angular Velocity	Maximum Flexion	Minimum Flexion
Affected vs. Unaffected Leg	0.2613	0.1333	0.6581	0.9445
Exercises	<0.0001	<0.0001	0.0885	<0.0001
Leg vs. Exercises	0.3807	0.1368	0.7262	0.9023
Time	0.3194	0.2521	0.9675	0.3826
Leg vs. Time	0.6637	0.0665	0.0747	0.8477
Exercises vs. Time	0.027	0.5549	0.2693	0.3152
Leg vs. Exercises vs. Time	0.2032	0.534	0.1465	0.2343

**Table 4:** Summary of three-way repeated measures ANOVA of ground-reaction force variables.

Kinetic Variables (*p* Values)			
Comparison	Peak Vertical Force (Z_Peak_)	Propulsion (Y_APeak_)	Braking (Y_BPeak_)
Affected vs. Unaffected Leg	0.7838	0.5418	0.4610
Exercises	<0.0001	0.0002	0.2631
Leg × Exercises	0.8394	0.9706	0.9144
Time	0.7132	0.2127	0.3422
Leg vs. Time	0.4063	0.3110	0.8742
Exercises × Time	0.0514	0.5281	0.9687
Leg × Exercises × Time	0.9915	0.7742	0.2417

## Data Availability

Data available on request.
